# Stimulus-hand proximity shapes conflict processing by task-dependent modulation of distractor activation

**DOI:** 10.1371/journal.pone.0361060

**Published:** 2026-11-03

**Authors:** Ruben Ellinghaus, Aldo Sommer, Rolf Ulrich, Rico Fischer, Roman Liepelt

**Affiliations:** 1 Chair of Cognitive Psychology: Judgment, Decision Making, Action, Faculty of Psychology, FernUniversität in Hagen, Hagen, Germany; 2 Department of Psychology, University of Greifswald, Greifswald, Germany; 3 Department of Psychology, University of Tübingen, Tübingen, Germany; Morsani College of Medicine, University of South Florida, UNITED STATES OF AMERICA

## Abstract

Placing the hands near stimuli modulates interference effects in conflict tasks. Liepelt and Fischer (2016) found that the direction of this near-hand effect depends on task demands: In a complex number-categorization Simon task, conflict was reduced when visual stimuli appeared near the hand, whereas in a simpler color-discrimination Simon task, conflict increased. They attributed this result pattern to a flexible attentional weighting of bottom-up vs. top-down processing. The present study specified and tested this hypothesis by applying the Diffusion Model for Conflict (DMC) to their data. The DMC assumes two parallel evidence accumulation processes: a transient distractor-based and a constant target-based diffusion process, which combine additively to drive response selection. Across two experiments, model fits revealed that stimulus-hand proximity selectively modulated the strength of distractor-based activation, while other parameters remained relatively stable across hand conditions. Crucially, this modulation mirrored the behavioral pattern: greater distractor activation near the hands in the color task producing increased conflict, but reduced activation near the hands in the number task leading to reduced conflict. These findings suggest that conflict is shaped by stimulus-hand proximity through selective adjustment of distractor-based processing, not target-based processing: location signals are typically amplified near the hands but down-regulated under higher cognitive demands. The results further illustrate how embodied manipulations can selectively impact specific processing components as mapped within a formal decision-making model.

## Introduction

Studies in embodied cognition demonstrated that cognitive processing can be modulated by stimulus-hand proximity [1–15]. This stimulus-hand proximity effect has been shown in experimental setups in which participants placed their hands on the response buttons located either close to the stimulus (proximal condition) or far from it (distal condition; see [1] Figure 1, for an illustration of this experimental setup). Within this line of research, a series of studies investigated how stimulus-hand proximity modulates cognitive processing in conflict tasks [2,4,7–9,11–13,15], in which irrelevant information slows down responses to task-relevant information. In particular, previous research reported reduced interference effects from task-irrelevant flankers [2], task-irrelevant semantic information in a Stroop task ([4], but see [9]), or between-task interference in a dual-task setting [7,15]. These findings suggest that placing the hands near the stimuli facilitates the response to task-relevant information while ignoring or inhibiting task-irrelevant (distracting) input.

**Fig 1:**
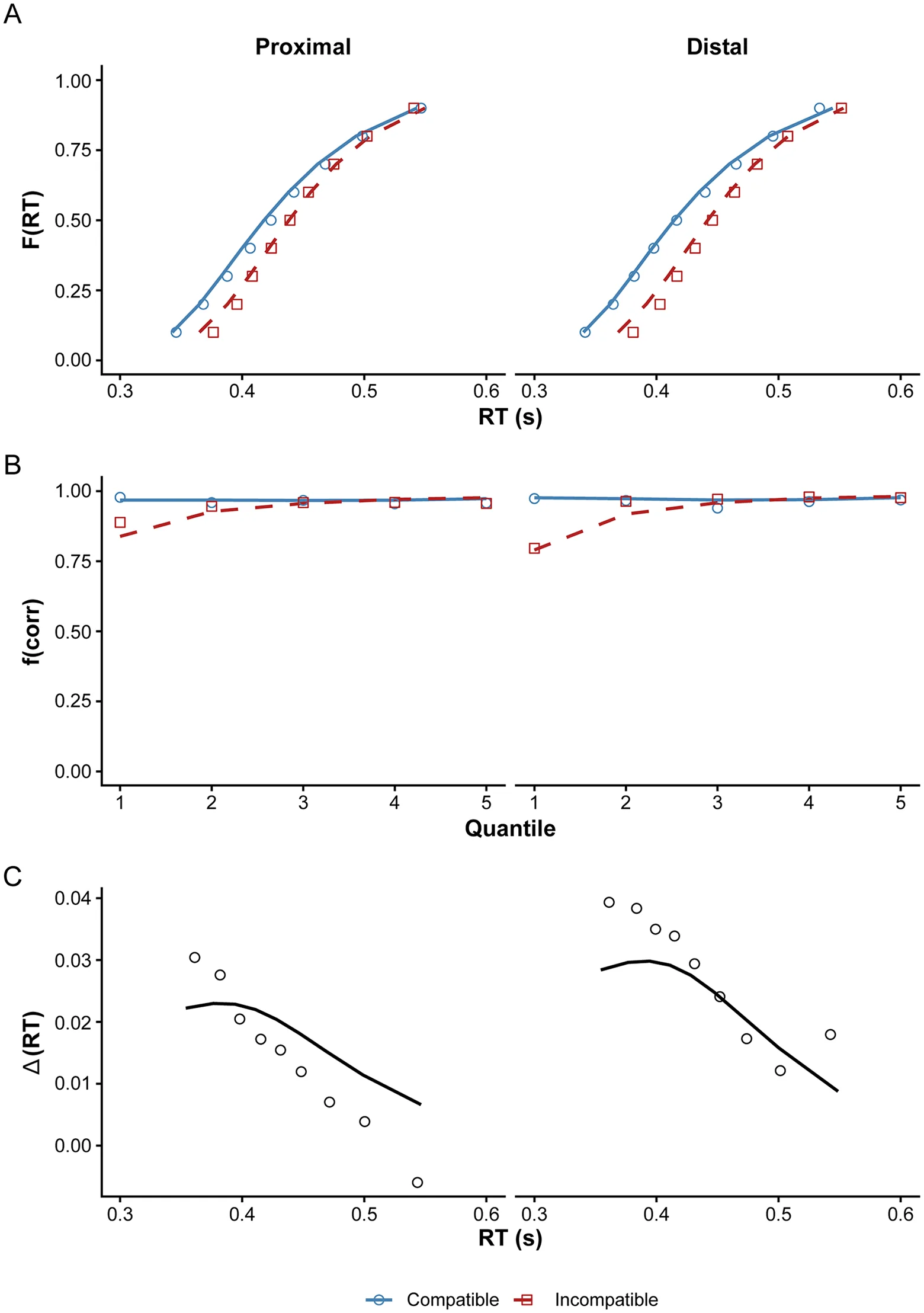
Experiment 1 – Empirical results (circles and rectangles) and DMC predictions of the best-fitting parameters (solid and dashed lines, respectively) for the proximal (left column) and distal (right column) Hand position. Panel A and B represent cumulative distribution functions and conditional accuracy functions as a function of Compatibility, respectively, and panel C represents RT delta plots.

However, this aforementioned conclusion is challenged by some inconsistent findings obtained with the Simon task. In this task, participants are asked to respond to the identity of a laterally presented stimulus while ignoring its location. Typically, interference from the task-irrelevant location is evident in slower and more error-prone responses when the required response is spatially incongruent with the stimulus location [16–18]. When responding to a perceptual feature like color, this Simon effect is often increased in the proximal compared to the distal stimulus-hand condition [8,9,11–14]. However, by increasing the task’s complexity, Liepelt and Fischer [8] reversed the direction of this near-hand effect. That is, in a Simon task where the participants judged the numerical category of numbers, they observed a reduced congruency effect in the proximal compared to the distal condition.

Liepelt and Fischer [8] explained the observed counter-directional modulation by adherence to traditional dual-route architectures [19–26]. These architectures propose that relevant and irrelevant information is processed in parallel via separate routes: the direct (automatic) route processes task-irrelevant information that overlaps with the response dimension (e.g., distractor-related information), while the indirect (controlled) route processes task-relevant target information according to the instructed stimulus-response mapping. Critically, while the indirect route requires top-down processing of the relevant information source, the distracting information can enter the direct route in a stimulus-driven (bottom-up) manner.

Most importantly, Liepelt and Fischer [8] speculated that these two types of processing are strategically modulated near the hands through flexible attentional weighting linked to task demands. In the applied number-judgement Simon paradigm [27] of the study, eight digits were mapped onto two responses via an arithmetic rule (smaller/larger than five), which is more complex compared to the one-to-one stimulus-response mapping applied in a color-discrimination Simon task, and in consequence more cognitively demanding. Specifically, under relatively low cognitive demands, bottom-up processing through the direct route may be enhanced in the proximal compared to the distal condition, whereas under relatively high cognitive demands, top-down control over stimulus processing may be enhanced in the proximal compared to the distal condition. Although this latter explanation is an important step toward clarifying the mechanisms underlying altered conflict processing in near-hand space, it leaves open the question of how, in detail, stimulus-hand proximity differentially modulates conflict processing as a function of task demands. Specifically, the notion of enhanced top-down processing may either translate to enhanced processing of target-related information (i.e., faster stimulus identification and selection of the instruction-based response; cf. [28–31], or to enhanced inhibition of distractor-based activation (e.g., [25,32–36]). One effective approach to test such fine-grained theoretical distinctions is to model the processes in formal, rather than purely verbal, terms. Mathematical process models establish a strong logical connection between latent processing components and behavioral performance. This precision makes behavioral data more informative regarding the underlying cognitive mechanisms than is typically possible with verbal theories (e.g., [37,38]).

In the present study, we took such a model-based approach by fitting the data of Liepelt and Fischer [8] to the Diffusion Model for Conflict (DMC; [39]). Diffusion models in general have been proven especially useful for analyzing performance in reaction time tasks [40,41]. According to these models, during speeded binary decisions, noisy evidence is accumulated over time between two boundaries, one for each response alternative. At each moment, steps toward the correct-response boundary are more likely, but random fluctuations may temporarily drive the evidence in the opposite direction. The average rate at which the evidence approaches the correct boundary is termed the drift rate. As soon as one boundary is reached, the corresponding response is initiated. This general architecture accounts for both variability in response times (due to noise, the path to the boundary is sometimes shorter, sometimes longer) and errors (noise occasionally drives the accumulation to the wrong boundary). Importantly, when such a model is fitted to observed response time and error data, its parameters can be estimated separately for different experimental conditions, revealing which component of the underlying decision process an experimental manipulation affects.

As further development, DMC situates the dual-route architecture introduced above within this diffusion modeling framework [39,40,42] and implements both the indirect and the direct route as diffusion processes. The target-based process of the indirect route reflects controlled processing: it accumulates evidence for the instructed response at a constant drift rate, $\nu_{c}$ (the controlled drift rate). The distractor-based process via the direct route, by contrast, is driven by automatic activation and has a time-dependent drift rate: In Simon tasks, the task-irrelevant stimulus location initially pushes evidence accumulation toward the response spatially associated with it, before this activation decays again. This pulse-like activation is described by its amplitude *A* (i.e., the strength of the activation) and by its time course *τ* (i.e., how quickly the pulse rises and decays). At any given time, the drift rates of the two processes are combined additively, and a response is triggered once the resulting superimposed process reaches one of the two decision boundaries, whose separation is defined by the boundary parameter *b*. Importantly, in compatible trials, the target-based process and the distractor-based process drive the accumulated evidence toward the same (i.e., the correct) boundary, such that the automatic activation facilitates the correct response. In contrast, in incompatible trials, the two processes drive the evidence toward opposite boundaries, resulting in the slower and more error-prone responses producing the typical compatibility effect.

Our hypotheses were guided by the flexible-weighting account given by Liepelt and Fischer [8], translated into the formal language of DMC. Recall that the flexible-weighting account of Liepelt and Fischer [8] predicts a task-dependent shift in the relative contributions of the direct and indirect processing routes near the hands but leaves open how this shift is realized mechanistically. Within DMC, it can come about either through changes in the controlled drift rate *ν*_*c*_ (altered efficiency of target-based processing) or through changes in the distractor amplitude *A* (altered strength of automatic activation). These two parameters thus instantiate the two candidate mechanisms and are therefore of primary interest. To provide a fair test, we additionally allowed the distractor time course *τ* and the boundary separation *b* to vary between conditions: Mittelstädt et al. [43] showed that counter-directional modulations of the compatibility effect between conflict tasks can in principle also arise from a codirectional modulation of *b*, as long as the tasks differ substantially in distractor timing *τ*. Had we instead held *τ* and *b* constant, any task-dependent difference would necessarily have been absorbed by *ν*_c_ and *A*, so that an effect on these two parameters would emerge almost by construction; allowing all four parameters to vary gives this plausible alternative a fair chance to account for the data.

For Experiment 1, which consisted of a number-categorization Simon task, we thus expected the attenuated Simon effect in the proximal compared to the distal condition to be reflected in a larger drift rate $\nu_{c}$ (reflecting enhanced target-based processing) and/or a smaller distractor amplitude *A* (reflecting attenuated distractor-based activation). For Experiment 2, which consisted of both a number-categorization and a color-discrimination Simon task, we expected to replicate the result of Experiment 1 for the number-categorization Simon task, but to observe the opposite pattern for the color-discrimination Simon task, thus mirroring the behavioral pattern: Smaller drift rate $\nu_{c}$ and/or larger distractor amplitude *A* for the proximal compared to the distal condition.

## Method

### Data Preparation

Preparation of the raw data was identical to the procedure reported by Liepelt and Fischer [8] for behavioral analysis. As in the original study, the main analysis was restricted to the data of the second block of each hand condition. Importantly, repeating the fitting procedure with the entire data set, no different conclusions were reached. Ethics approval, consent to participate and for publication were not required, as the present study involved a re-analysis of previously collected, anonymized data and no new data were collected.

### Fitting Procedure

We fitted the prepared data from both experiments to the DMC. As guided by our hypotheses, the controlled drift rate $\nu_{c}$, as well as τ, A, and *b* were allowed to vary as a function of Hand position (proximal vs. distal) in Experiment 1 and as a function of both, Hand position (proximal vs. distal) and Task (number vs. color) in Experiment 2. We estimated the remaining model parameters (e.g., parameters reflecting the shape of automatic activation or non-decision time) across conditions. These models were fitted to individual reaction time (RT) and accuracy data via full-distribution maximum likelihood methods using the R package dRiftDM [44]. Optimization was done via differential evolution [45]. The diffusion constant, which scales the model, was set to *σ* = 1. Evidence values and time were discretized in steps of 0.002.

### Data Analysis

The best-fitting parameters were compared between the conditions (Hand position: proximal, distal) via paired *t*-test (one-sided for $\nu_{c}$ and *A*, two-sided for τ and *b*) for the analysis of Experiment 1. Model parameters of Experiment 2 were analyzed by a 2 (Hand position: proximal, distal) × 2 (Task: color, number) within-subjects repeated measures analysis of variance (rmANOVA), and additional paired comparisons between hand positions in each task (one-sided for *A*, two-sided for the remaining parameters). Descriptive data are reported as the arithmetic mean and standard error.

## Results & Discussion

### Experiment 1

An overview of the modelling results for Experiment 1 (number-categorization Simon task) is given in Fig 1. Visual inspection suggests that the estimated parameters provide a good fit to the data. The observed delta plots and conditional accuracy functions (CAFs) were negative-going, characteristic of the Simon task, and the model predictions closely tracked them, consistent with the pulse-like, transient distractor activation assumed by the DMC (see Fig 1 panels B and C).

Table 1 compares the hand conditions for each task and parameter. Most importantly, the amplitude *A* of automatic activation was significantly reduced for the proximal compared to the distal condition, *t*(19) = −2.84, *p* = .005, *d* = 0.64, while the remaining parameters did not differ meaningfully between conditions (all *p* > .573). In essence, our DMC-based analysis suggests that selective attenuation of distractor-based activation strength, rather than amplification of target-based processing, drove the reduced Simon effect observed in the behavioral data of the number-categorization Simon task.

**Table 1:** Best-Fitting Parameters for Experiment 1. As guided by our hypotheses, *t*-tests are one-sided for distractor amplitude (*A*) and controlled drift rate ($\nu_{c}$) but two-sided for the remaining parameters.

Parameter	Distal	Proximal	*t(*df)	*p*	*d*
Distractor amplitude (*A*)	*M* = 0.14, *SE* = 0.01	*M* = 0.11, *SE* = 0.01	*t*(19) = −2.84	.005	0.64
Boundary separation (*b*)	*M* = 0.51, *SE* = 0.03	*M* = 0.52, *SE* = 0.03	*t*(19) = 0.16	.873	0.04
Controlled drift rate ($\nu_{c}$)	*M* = 4.71, *SE* = 0.22	*M* = 4.68, *SE* = 0.23	*t*(19) = −0.17	.566	0.04
Distractor timing (*τ*)	*M* = 0.05, *SE* = 0.01	*M* = 0.04, *SE* = 0.01	*t*(19) = −0.57	.573	0.13

### Experiment 2

An overview of the modelling results for Experiment 2 is given in Fig 2. Visual inspection suggests that the estimated parameters again provide an acceptable fit to the data. As in Experiment 1, the observed delta functions were negative-going and were closely tracked by the model predictions (see Fig 2 panel B and C). Table 2 compares the hand conditions across tasks and parameters.

**Table 2:** Best-Fitting Parameters for Experiment 2. As guided by our hypotheses, *t*-tests are one-sided for distractor amplitude (*A*) but two-sided for the remaining parameters.

Parameter	Task	Distal	Proximal	*t*(df)	*p*	*d*
Distractor amplitude (*A*)	color	*M* = 0.11, *SE* = 0.01	*M* = 0.13, *SE* = 0.01	*t*(54) = 1.47	.073	0.20
	number	*M* = 0.13, *SE* = 0.01	*M* = 0.11, *SE* = 0.01	*t*(54) = −1.40	.083	0.19
Boundary separation (*b*)	color	*M* = 0.49, *SE* = 0.02	*M* = 0.51, *SE* = 0.02	*t*(54) = 1.55	.127	0.21
	number	*M* = 0.62, *SE* = 0.02	*M* = 0.62, *SE* = 0.02	*t*(54) = −0.25	.800	0.03
Controlled drift rate ($\nu_{c}$)	color	*M* = 3.77, *SE* = 0.16	*M* = 3.94, *SE* = 0.14	*t*(54) = 1.26	.212	0.17
	number	*M* = 3.96, *SE* = 0.13	*M* = 4.07, *SE* = 0.13	*t*(54) = 0.85	.402	0.11
Distractor timing (*τ*)	color	*M* = 0.04, *SE* = 0.00	*M* = 0.06, *SE* = 0.01	*t*(54) = 1.73	.090	0.23
	number	*M* = 0.07, *SE* = 0.01	*M* = 0.07, *SE* = 0.01	*t*(54) = −0.23	.823	0.03

**Fig 2:**
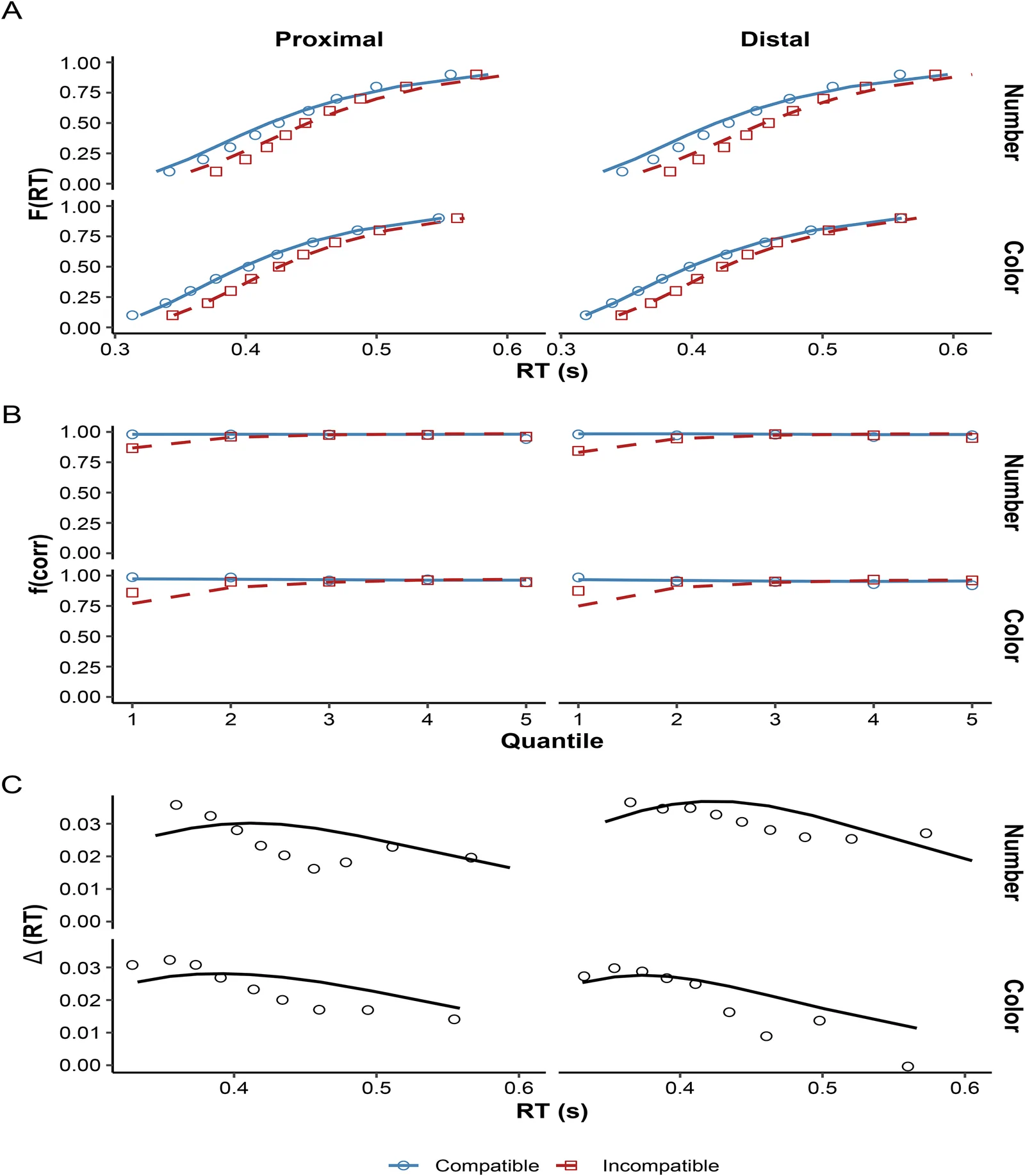
Experiment 2 – Empirical results (circles and rectangles) and DMC predictions of the best-fitting parameters (solid and dashed lines). Each panel represents the proximal (left) alongside the distal (right) Hand position. Panel A. Cumulative distribution functions of correct RTs as a function of Compatibility and Task (color vs number). Panel B. Conditional accuracy functions (CAFs) as a function of Compatibility and Task. Panel C. RT delta plots of the Simon effect as a function of task.

For peak time *τ*, the rmANOVA revealed no significant main effect of Hand position, *F*(1, 54) = 1.13, *p* = .293, *η²* = .02, but a significant main effect of Task, *F*(1, 54) = 6.50, *p* = .014, *η²* = .11, reflecting overall larger *τ* (*M* = 0.07) in the number task compared to the color task (*M* = 0.05). This result replicates earlier evidence suggesting that the peak time of distractor-based activation varies substantially between different variants of conflict tasks [29,39]. The interaction between Hand position and Task was not significant, *F*(1, 54) = 2.50, *p* = .119, *η²* = .04.

For controlled drift rate ($\nu_{c}$), the rmANOVA revealed no significant main effects of Hand position, *F*(1, 54) = 1.89, *p* = .175, *η²* = .03, or Task, *F*(1, 54) = 1.72, *p* = .195, *η²* = .03. The interaction between Hand position and Task was not significant, *F*(1, 54) = 0.13, *p* = .716, *η²* = .00.

For boundary separation (*b*), the rmANOVA revealed no significant main effect of Hand position, *F*(1, 54) = 0.43, *p* = .515, *η²* = .01, but a significant main effect of Task, *F*(1, 54) = 61.68, *p* < .001, *η²* = .53, indicating that decision boundaries were higher in the number task (*M* = 0.62) compared to the color task (*M* = 0.50). This suggests that participants prioritized accuracy over speed more in the former than in the latter case [43]. However, the interaction between Hand position and Task was not significant, *F*(1, 54) = 1.43, *p* = .237, *η²* = .03.

For distractor amplitude (*A*), the rmANOVA revealed no significant main effect of Hand position, *F*(1, 54) = 0.02, *p* = .902, *η²* = .00. Similarly, there was no significant main effect of Task, *F*(1, 54) = 0.00, *p* = .965, *η²* = .00. Most importantly, however, the interaction between Hand position and Task was significant, *F*(1, 54) = 4.57, *p* = .037, *η²* = .08 (depicted in Fig 3), indicating a reversed pattern in how stimulus-hand proximity modulated the task-specific Simon effects, replicating Liepelt and Fischer [8]. In the number task, the distractor amplitude was numerically reduced in the proximal (*M* = 0.11) compared to the distal (*M* = 0.13) condition as in Experiment 1, whereas the opposite pattern with numerically increased A in the proximal (*M* = 0.13) compared to the distal (*M* = 0.11) condition was found for the color task. Both post-hoc tests, however, did not reach statistical significance, with *t*(54) = –1.40, *p* = .083, *d* = 0.19 (one-sided) for the number task and *t*(54) = 1.47, *p* = .073, *d* = 0.20 (one-sided) for the color task.

**Fig 3:**
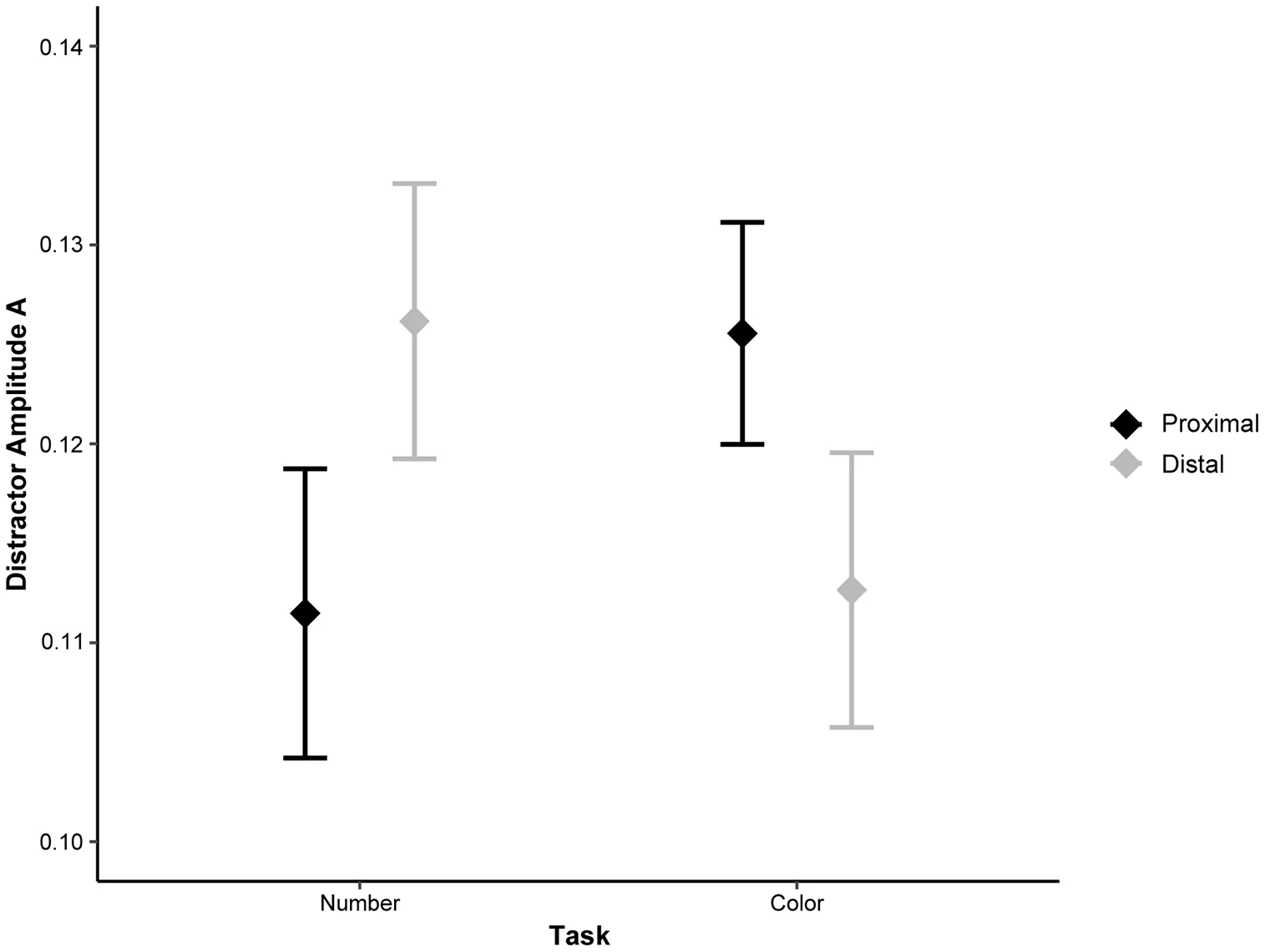
Experiment 2 – Distractor activation strength (amplitude *A*) as a function of Task (number vs. color) and Hand position (proximal vs. distal). Error bars reflect SEM after Morey [46].

## General discussion

Liepelt and Fischer [8] reported larger spatial compatibility effects in the proximal than distal hand condition for a color-discrimination Simon task, but the reverse pattern for a cognitively more demanding number-categorization Simon task. They attributed this reversal to a flexible weighting of bottom-up vs. top-down processing depending on the task’s demands. What remained unclear, however, was which processing components the stimulus-hand proximity actually altered under different task demands. Using DMC [39] to re-analyze their data, we found that stimulus-hand proximity selectively modulates the strength of distractor-based activation (amplitude *A*), whereas target-based processing drift $\nu_{c}$, boundary separation *b*, and the distractor-related time course *τ* were largely unaffected by hand position. Critically, the direction of the *A* modulation mirrored the behavioral pattern: in the color task, *A* was larger near the hands, explaining increased conflict; in the number task, *A* was smaller near the hands, explaining decreased conflict. In addition, *b* and *τ* were higher in the number task than in the color task, indicating more cautious processing and a slower build-up of distractor activation in the number task.

Taken together, our results suggest that stimulus-hand proximity modulates conflict processing by selectively influencing distractor-based activation caused by the irrelevant location information rather than target-related processing. Importantly, localizing the effect of stimulus-hand proximity on the distractor amplitude *A* rather than on the controlled drift rate *ν*_*c*_ is fully consistent with a cognitive-control account. The direct route is initiated automatically, but its strength is not immune to top-down control: amplitude *A* reflects the effective strength of distractor-based activation, which can be up- or down-regulated via adaptive control through top-down suppression [34]. In line with previous studies [9,11–14], the default tendency appears to be amplification of distractor (location) processing in the proximal compared to the distal condition, which, however, can be strategically down-regulated as the system allocates more top-down control [47]. The observed directional differences of the stimulus-hand proximity effect between the Simon task variants reported by Liepelt and Fischer [8] fit this account once task-specific demands and timing are considered: in a relatively easy/fast task (e.g., color discrimination), decisions terminate while the brief, pulse-like distractor activation is still strongly influential, so the default amplification of *A* near the hands effectively increases interference. In a more demanding/slower task (e.g., numerical categorization), participants adopt more cautious settings and have more time for gradual top-down suppression to counteract the initial amplification (cf. [35,36,48–50]), effectively reducing the overall strength of distractor activation. Recent neurophysiological evidence on sequential dependencies in conflict tasks (cf. [20]) supports this selective suppression account of cognitive control adjustments; Lee, Bae and Cho [51] found reduced location decoding in the high beta band after incongruent compared to congruent trials in a Simon task, while no difference in decoding accuracy was observed for target representations.

It is presently unclear whether the task-specific effects of stimulus-hand proximity on inhibitory control are mediated solely via demand-related timing differences. An additional relevant factor might be intentional weighting [52]: before stimulus presentation, the task set assigns greater weight to goal-relevant feature dimensions and down-weights irrelevant ones via a top-down signal. In the number task, the goal-relevant feature is semantic, and hence low-level perceptual dimensions – including location – should be down-regulated. By contrast, in the color task, the goal-relevant feature (color) itself belongs to a low-level perceptual dimension, so related low-level features such as location might not be downregulated substantially. Given that placing the hands near the stimuli might generally strengthen top-down control factors (e.g., [2]) the difference between the tasks in the relative intentional weighting of relevant vs. irrelevant information might be further amplified in the proximal compared to the distal condition.

As a general limitation of our approach, it should be noted that our modelling isolates only decisional processing stages but does not capture early sensory and late motor stages, which might also contribute to the altered processing of spatial information as a function of hand position (e.g., [2]). However, conclusive evidence places the Simon effect in post-perceptual stages (e.g., [36,50,53,54]), and the modulation of the Simon effect by stimulus-hand proximity has also been attributed to later processing stages [13].

In conclusion, the present contribution indicates that embodied manipulation (stimulus-hand proximity) exerts a component-specific influence on conflict processing: it modulates distractor-based activation while leaving target processing and response caution largely intact. Taken together, the results support theories of flexible, task-demand-dependent control and further demonstrate that embodied influences can be precisely mapped within formal mechanistic models.
